# Rabies in Ferret Badgers, Southeastern China

**DOI:** 10.3201/eid1506.081485

**Published:** 2009-06

**Authors:** Shoufeng Zhang, Qing Tang, Xianfu Wu, Ye Liu, Fei Zhang, Charles E. Rupprecht, Rongliang Hu

**Affiliations:** Academy of Military Medical Sciences, Changchun, People’s Republic of China (S. Zhang, Y. Liu, F. Zhang, R. Hu); Chinese Center for Disease Control and Prevention, Beijing, People’s Republic of China (Q. Tang); Centers for Disease Control and Prevention, Atlanta, Georgia, USA (X. Wu, C.E. Rupprecht)

**Keywords:** Rabies, ferret badgers, rabies virus, retrospective epidemiological surveillance, spillover, viruses, China, dispatch

## Abstract

Ferret badger–associated human rabies cases emerged in China in 1994. We used a retrospective epidemiologic survey, virus isolation, laboratory diagnosis, and nucleotide sequencing to document its reemergence in 2002–2008. Whether the cause is spillover from infected dogs or recent host shift and new reservoir establishment requires further investigation.

Rabies is an acute encephalomyelitis caused by rabies or rabies-related viruses. Although dogs are the main reservoir worldwide, all mammals are believed to be susceptible. When rabies is widely distributed, affected wildlife may constitute a public health threat to local residents. For example, the Chinese ferret badger (*Melogale moschata*) has been associated with human rabies for several years, although diagnoses have not been confirmed (*1*–*4*). Rabies has also been reported in other subspecies, such as honey badgers (*Mellivora capensis*) and European badgers (*Meles meles*) in Africa and Europe. Transmission was presumed to occur independently among the population or as spillover from other reservoirs, such as jackals, dogs, or foxes (*5*,*6*). However, none of these animals have been reported to be associated with human deaths. The Chinese ferret badger, which dwells mainly in southeastern China, is a different subspecies than the badgers in Africa and Europe. These mustelids have several names in southern China—crab-eating mongoose, rice field dog, viviparid-eating dog, loach-eating dog, and white face weasel—mainly because of their omnivorous behavior and external appearance. Recently, human rabies associated with Chinese ferret badgers has seemed to reemerge.

Because the People’s Republic of China has no governmental surveillance network, few data exist on wildlife rabies in China, and therefore the natural behavior and habitats of Chinese ferret badgers are not clear (*7*). Most background information about this animal species in this report was obtained from local hunters. Chinese ferret badgers are solitary and nocturnal. Those observed during daylight are usually sick. The animals are distributed widely in China but are concentrated mainly in Anhui, Zhejiang, and Jiangxi provinces (Figure 1). However, the detailed population density of the badgers is largely unknown.

**Figure 1:**
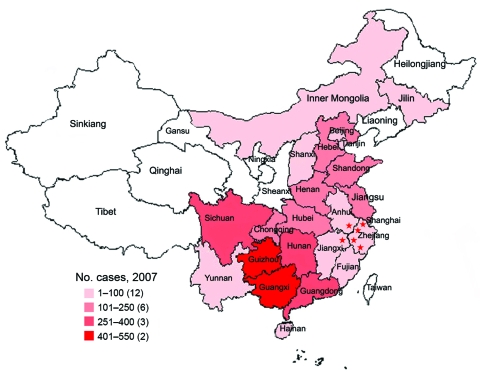
Distribution of human rabies cases in mainland China, 2007. Red stars indicate ferret badger–associated human rabies cases. Numbers in parentheses in key indicate number of affected provinces.

Ferret badger–associated human rabies cases in China were first reported in 1997 but had actually emerged in 1994 (*1*). During that year, 6 patients with clinical signs of rabies received a preliminary diagnosis at Huzhou Second Hospital, Huzhou District, Zhejiang. In 1995, a similar case was reported in the same hospital. Among the 7 case-patients, 6 were reported to have been bitten on the hands by ferret badgers. This could be the first alleged epizootic of ferret badger–associated human rabies. From 1999 through 2003, 4 ferret badger–associated human rabies cases were reported in Huzhou, and 14 cases were reported in Hangzhou (*8*,*9*), the capital district of Zhejiang. In 2004, 1 human case in Huzhou and 3 human cases in Hangzhou were recorded (*10*,*11*). From 1994 through 2004, 12 (60%) of 20 human rabies cases in Huzhou, and 17 (77%) of 22 human rabies cases in Hangzhou were associated with ferret badger exposure. Ferret badger–associated human rabies in the western counties of Hangzhou were frequently reported in local news (http://zjajcdcsy.zjwst.gov.cn/col71/info.htm1?infoid=605, http://news.sina.com.cn/c/2003-07-08/09391300011.shtml, www.zj.xinhua.org/old/200212/4/100021681.htm, and www.jksoso.com/html/0F1A6B60.htm).

In Jing County, which is located in eastern Anhui and is adjacent to the western border of Zhejiang, 3 human rabies cases associated with ferret badger bites were reported successively in 1999, 2000, and 2001 (*4*,*12*). An incorrect photograph of the ferret badger was cited in a previous brief report (*3*).

To determine whether ferret badger–associated rabies is reemerging in China, we conducted a retrospective epidemiologic survey in the affected regions from 2002 through 2008. To document ferret badger–associated rabies, we used virus isolation, laboratory diagnosis, and nucleotide sequencing.

## The Study

During 2002–2004, many sick badgers were seen at the bases of mountains, on village roads, and within residential houses. At the same time, rabies in livestock was reported in the nearby villages. Concomitantly, the highest number of human rabies cases was recorded during that period. Local residents stated that dead animals were seen everywhere; however, accurate numbers and distribution of affected animals in these areas were difficult to estimate.

During 2005–2007, ferret badger hunters were recruited to help capture the animals for further investigation; 1–2 badgers were captured each week. The badgers were no longer commonly seen in the fields, probably the result of depopulation by the disease. Among the 58 specimens collected in Lin’an, Chun’an, and Jiande counties of Hangzhou, none of the brain tissue samples were positive for rabies by standard direct fluorescent assay. Serum samples from the 63 animals captured in the 3 counties mentioned above did not have detectable rabies virus–neutralizing antibodies according to the fluorescent antibody virus neutralization test (Table 1).

**Table 1:** Rabies fluorescent antibody virus neutralization assay results of ferret badger serum samples, China

Date	No. samples (no. positive)	IU/mL*
2005–2007	63 (0)	0
Apr–Jul 2008	30 (5)	0.20, 0.33, 0.45, 0.5, 0.8

*Arbitrary cut-off value for seroconversion is 0 IU/mL.

During 2007–2008, the population of the ferret badgers in the same regions seemed to recover, and rabies infection in badgers began to increase. Since the summer of 2008, sick and dead badgers have been seen by local residents inside houses, in the fields close to the residential areas, and on the roads in Hangzhou District. Of the 71 brain samples collected in 2008, 4 had positive direct fluorescent assay results. Of 30 serum samples, 5 had positive results for rabies virus–neutralizing antibody (Table 1). In addition, a human rabies case was recorded in April 2008 in Lishui County, Zhejiang. Our most recent retrospective epidemiologic investigation of human rabies cases from the end of 2007 through 2008 showed that in Wuyuan County, Jiangxi, adjacent to Hangzhou, Zhejiang, 4 of 5 recorded human rabies cases were caused by badger bites.

**Figure 2:**
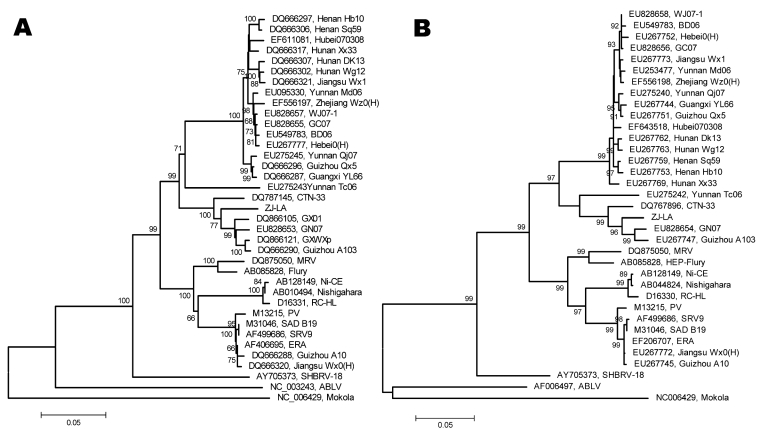
Position of a ferret badger–associated rabies virus isolate (ZJ-LA) in a phylogenetic tree constructed based on the nucleoprotein genes from representative dog rabies virus isolates and common vaccine strains in China (A) and the glycoprotein genes from representative dog rabies virus isolates and common vaccine strains in China (B). This figure was drawn by MEGA 4 (www.megasoftware.net) with maximum composite likelihood model. Bootstrap values are calculated from 1,000 repetitions. Scale bars represent phylogenetic distance between isolates.

Phylogenetic analysis using the nucleoprotein and the glycoprotein genes (Table 2) demonstrated that the ferret badger rabies virus isolate (ZJ-LA, isolated from a badger in Lin’an County of Hangzhou, Zhejiang) had 89.0% homology with a local dog rabies virus isolate (Zhejiang Wz0) and overall 86.5%–95.9% homology with other isolates from China (Figure 2). The ZJ-LA strain had the highest homology with a dog rabies virus isolate (GN 07, from Guangning County, Guangdong Province) and a vaccine strain CTN-33 (originally from a person who died of rabies in Ji’nan, Shandong Province, in 1957). Because dog-associated human rabies has been reported only sporadically in Zhejiang Province, whether the ferret badger–associated rabies is a spillover event from dogs, or the animals now serve as a natural reservoir in the rabies-endemic area, needs further investigation.

**Table 2:** Rabies virus isolates or strains used to construct phylogenetic tree (Figure 2)*

Isolate or strain	GenBank accession no.		Region of origin	Host	Year isolated
	Nucleoprotein	Glycoprotein			
BD06	EU549783	EU549783	Hebei	Dog	2006
CTN-33	DQ787145	DQ767896	Shangdong	Dog	1957
GC07	EU828655	EU828656	Hebei	Dog	2007
GN07	EU828653	EU828654	Guangdong	Dog	2007
Guangxi_YL66	DQ666287	EU267744	Guangxi	Dog	2006
Guizhou_A10	DQ666288	EU267745	Guizhou	Human	2004
Guizhou_A103	DQ666290	EU267747	Guizhou	Dog	2004
Guizhou_Qx5	DQ666296	EU267751	Guizhou	Dog	2004
GX01	DQ866105	NA	Guangxi	Dog	2006
GXWXp	DQ866121	NA	Guangxi	Dog	2006
Hebei0(H)	EU267777	EU267752	Hebei	Human	2007
Henan_Hb10	DQ666297	EU267753	Henan	Dog	2004
Henan_Sq59	DQ666306	EU267759	Henan	Dog	2004
Hubei070308	EF611081	EF643518	Hubei	Buffalo	2007
Hunan_DK13	DQ666307	EU267762	Hunan	Dog	2004
Hunan_Wg12	DQ666308	EU267763	Hunan	Dog	2004
Hunan_Xx33	DQ666317	EU267769	Hunan	Dog	2004
Jiangsu_Wx1-06	DQ666321	EU267773	Jiangsu	Dog	2004
Jiangsu_Wx0(H)	DQ666320	EU267772	Jiangsu	Human	2004
MRV	DQ875050	DQ875050	Henan	Mouse	1987
WJ07-1	EU828657	EU828658	Hebei	Dog	2007
Yunnan_Md06	EU095330	EU253477	Yunnan	Dog	2006
Yunnan_Qj07	EU275245	EU275240	Yunnan	Dog	2007
Yunnan_Tc06	EU275243	EU275242	Yunnan	Dog	2006
Zhejiang Wz0(H)	EF556197	EF556198	Zhejiang	Human	2007
**ZJ-LA**	**FJ598135**	**FJ719756**	**Zhejiang**	**Ferret badger**	**2008**
ABLV	NC003243	AF006497	Australia	Bat	1996
ERA	AF406695	EF206707	France	Vaccine strain	2003
HEP-Flury	AB085828	AB085828	Japan	Vaccine strain	2003
Mokola	NC006429	NC006429	France/USA	Bat	1997
Nishigahara	AB010494	AB044824	Japan	Vaccine strain	1998
Ni-CE	AB128149	AB128149	Japan	Vaccine strain	2007
PV	M13215	M13215	France	Vaccine strain	1993
RC-HL	D16331	D16330	Japan	Vaccine strain	1994
SAD-B19	M31046	M31046	USA	Vaccine strain	1990
SHBRV-18	AY705373	AY705373	USA	Bat	1996
SRV9	AF499686	AF499686	Clone of SAD-B19	Vaccine strain	2006

*NA, not available; **boldface** indicates the isolate reported in this article.

## Conclusions

Rabies in ferret badgers occurred during 2 alleged epizootics (1994–1995 and 2002–2004) in southeastern China (Figure 1) (*13*). Our preliminary data suggest another probable epizootic of rabies in ferret badgers during 2007–2008. Rabies in ferret badgers is becoming a greater public health threat to humans in eastern Anhui, middle to western Zhejiang, and northern Jiangxi provinces in China.

Because no practical rabies vaccine has been developed for wildlife in China, a rabies epidemic in ferret badgers is almost inevitable without intervention, and the threat to public health is immediate. Lack of communication and cooperation among the Chinese Center for Disease Control and Prevention, Ministry of Agriculture, and wildlife services from the Bureau of Forestry makes the situation more complicated than canine rabies control. Whether rabies in ferret badgers is a spillover event from rabid dogs or whether ferret badgers serve as a natural reservoir remains to be addressed. In addition to more detailed epidemiologic investigations, control and elimination of rabies in dogs is a primary suggestion to test the latter hypothesis.
